# miR-199a-5p inhibits malignant progression and enhances cisplatin sensitivity of nasopharyngeal carcinoma by targeting SLC1A5

**DOI:** 10.1016/j.bjorl.2025.101739

**Published:** 2025-11-28

**Authors:** Anchi Sun, Rongrong Lv, Zhiwei Xing, Yifan He, Xiaomin Wang, Hui Li, Qianqian Shao

**Affiliations:** a Bengbu Medical University, Graduate School, Bengbu, China; b The Forth Affiliated Hospital of Anhui Medical University, Department of Otolaryngology, Hefei, China; c Anhui Engineering Technology Research Center of Biochemical Pharmaceutical, Bengbu, China; d The First Affiliated Hospital of Bengbu Medical College, Department of Otolaryngology, Bengbu, China; e Bengbu Medical University, School of Basic Medicine, Department of Human Anatomy, Bengbu, China; f Bengbu Medical University, Key Laboratory of Digital Medicine and Smart Health, Bengbu, China; g Jiading Branch, Ren Ji Hospital, Shanghai Jiao Tong University School of Medicine, Department of Otolaryngology, Shanghai, China

**Keywords:** miR-199a-5p, SLC1A5, Nasopharyngeal cancer, Cisplatin resistance, EMT

## Abstract

**Objective:**

The prevalence of Nasopharyngeal Carcinoma (NPC) is increasing, and the mortality of patients with cisplatin resistance is high. In this study, we aimed to elucidate several potential mechanisms of miR-199a-5p in regulating the progression and cisplatin resistance of NPC.

**Methods:**

Relevant datasets were downloaded from the Gene Expression Omnibus (GEO) database to verify miR-199a-5p expression in NPC. The miR-199a-5p level was evaluated by RT-qPCR, and the prognostic significance was analyzed by K–M curve. Cell proliferation, migration, invasion and apoptosis were detected in vitro. The contents of Epithelial-Mesenchymal Transition (EMT) protein, apoptosis protein and SLC1A5 protein were detected by Western immunoblotting. DLR assay elucidated that miR-199a-5p targets SLC1A5. The effect of miR-199a-5p on subcutaneous tumor in nude mice was detected in vivo.

**Results:**

We found that miR-199a-5p was differentially expressed in nasopharyngeal carcinoma, and patients with high expression showed good prognosis. It was also found to be associated with cisplatin resistance and showed low expression in HNE1/DDP cells. The up-regulation of miR-199a-5p can inhibit the proliferation, migration and invasion of NPC cells, and enhance cell apoptosis. The mechanism may be achieved by targeting SLC1A5.

**Conclusion:**

The high expression of miR-199a-5p can inhibit SLC1A5 and thus the progression of NPC. At the same time, the high expression of miR-199a-5p can increase the sensitivity of NPC to cisplatin.

**Level of evidence:**

Level 5.

## Highlights

• MiR-199a-5p was lowly expressed in NPC.
• Low expression of miR-199a-5p is associated with a poorer prognosis.
• MiR-199 can increase the sensitivity of NPC to cisplatin resistance.
• MiR-199a-5p directly targets SLC1A5 to negatively regulate its expression.

## Introduction

NPC is a relatively common tumor with a global incidence, ranking first in the incidence of head and neck surgical malignant tumors in southern China (>20/100,000 people), with male patients being 2–3 times more common than female patients.^1^ Cisplatin (DDP) based chemoradiotherapy has long been regarded as an effective means of treating NPC. However, the toxic side effects of cisplatin and the emergence of drug resistance continue to present a significant challenge for clinicians and patients alike.^2^

MicroRNAs (miRs), which are short non-coding RNAs, are associated with a variety of cancers and can be used as oncogenes or tumor suppressors.^3^^,^^4^ In previous studies, we found that miR-199a-5p presents differential expression in NPC, and previous studies have shown that miR-199a-5p is a potential regulator to inhibit NPC progression.^5^ Multiple studies have shown that miRs are involved in regulating cisplatin resistance.^6^^,^^7^ Recent studies have shown that miR-199a-5p may be related to drug resistance and has been confirmed to be related to doxorubicin resistance.^8^

In this study, we employed a comparison between the drug-resistant cell line HNE1/DDP and the parental cell line HNE1 to investigate the impact of miR-199a-5p on the proliferation, invasion, and migration of cisplatin-resistant nasopharyngeal carcinoma cells, along with its underlying mechanism. This research aims to offer novel insights and methodologies for treating and studying drug-resistant patients with nasopharyngeal carcinoma.

## Methods

### Main materials

DDP, Radio Immunoprecipitation Assay (RIPA) lysis buffer, CCK-8 (Cell Counting Kit-8) kit were procured from Beyotime Biotechnology Co., Ltd. RPMI (Roswell Park Memorial Institute) 1640 medium was acquired from Wuhan Sewell Biotechnology Co., Ltd. miR-199a-5p mimics, inhibitors, and negative control reagents were obtained from Huzhou Hippo Biotechnology Co., Ltd (Table 1). The Lipofectamine™ 2000 Kit and Trizol Reagent were purchased from Invitrogen, Inc. miR-199a-5p primers and U6 were designed and synthesized by Sangon Bioengineering (Shanghai) Co., Ltd (Table 2). The MonAmp miRNA Universal Super Specificity qPCR Mix Reverse Transcription Kit and MonScript™ miRNA First Strand cDNA Synthesis Kit (Tailing Reaction) were acquired from Mona (Suzhou) Biotechnology Co., Ltd. Matrigel was sourced from BD in the United States. Transwell chamber was obtained from Corning, Inc., USA. The monoclonal antibody SLC1A5, E-cadherin, N-cadherin, vimentin, GAPDH, and goat anti-rabbit IgG Horseradish Peroxidase (HRP) antibodies were purchased from Jiangsu Affinity Biological Research Center Co., Ltd. The Annexin V-FITC/PI double-stained apoptosis detection kit was acquired from Bestbio.

**Table 1:** Transfected gene sequences.

Gene	Sequence (5′→3′)
has-miR-199a-5p inhibitor	GAACAGGUAGUCUGAACACUGGG
Inhibitor NC	CAGUACUUUUGUGUAGUACAA
hsa-miR-199a-5p mimics	Sense CCCAGUGUUCAGACUACCUGUUC
	Antisense ACAGGUAGUCUGAACACUGGGUU
NC	Snse UUCUCCGAACGUGUCACGUTT
	Antisense ACGUGACACGUUCGGAGAATT

**Table 2:** Primer sequences used for qRT-PCR.

Gene	Primer sequence (5′→3′)
miR-199a-5p Forward	GCCCAGTGTTCAGACTACCTGTTC
U6-Forward	CTCGCTTCGGCAGCACA
U6-Reverse	AACGCTTCACGAATTTGCGT
GAPDH-Forward	GGAGCGAGATCCCTCCAAAAT
GAPDH-Reverse	GGCTGTTGTCATACTTCTCATGG
SLC1A5-Forward	ATGGAGCGGCGGCGCTGCTG
SLC1A5-Reverse	TCAGGGTCAGGAGGCTGAGG

The 3′ end primer was the Universal-GPCR-3 primer provided in the miRNA first-strand cDNA Synthesis kit (MR05301S) from Monad.

### Cells and treatments

Human HNE1 and HNE1/DDP cells were acquired from School of Pharmacy, Bengbu Medical University (Anhui, China), stored at −80 °C, thawed, and cultured with RPMI-1640 containing 10% FBS and penicillin/streptomycin in a CO_2_ incubator. Additionally, the culture medium of HNE1/DDP cells was supplemented with cisplatin at a concentration of 1 μg/mL.^9^

Lipofectamine™ 2000 (Invitrogen, USA) was used as directed for transfection with miR-199a-5p mimic, inhibitor, or blank control constructs. Experiments were performed after 48 h.

### Proliferation assessments

Proliferation was examined using CCK-8 assays. Cells plated in 96-well plates (5000 well) were cultured for 1–3 days, after which CCK-8 reagent (10 μL/well) was introduced for 2 h at 37 °C followed by the reading of absorbances (450 nm) with a microplate reader (Infinite F50; Tecan).

Colony formation assays were conducted by seeding cells in 6-well plates (1000 well) and culturing them for 14-days, after which they were fixed (4% paraformaldehyde, 20 min), stained for 20 min using crystal violet, rinsed using ddH_2_O, and imaged via microscopy. ImageJ (NIH, MD, USA) was used for image analysis.

### Migration assays

For wound healing assays, cells were cultured in 6-well plates (2 × 10^5^/well) until 90% confluent. Scratches were made in the monolayer with a 200 μL pipette tip. Imaging was then performed via microscopy after 0 and 24 h, and the resultant images were used to compute the rate of cellular migration.

Migration was measured using Transwell assay. 200 μL serum-free cell suspension was added to the upper part of the 24-well plate cell chamber (1 × 10^4^/well), and 600 μL serum-containing medium was added to the lower chamber. After 24 h of culture, the suspension in upper chamber was discarded, while the cells were fixed with 4% paraformaldehyde, then stained with crystal violet, imaged and counted by microscope, and three randomly selected areas were counted.

### Invasion experiments

Matrigel was added to the Transwell chambers before cell culture, and the other steps were the same as Transwell detection migration.

### Western immunoblotting (WB)

The total protein was obtained by lysing the cells with RIPA reagent, and its concentration was determined by the BCA method. The protein sample (30 μg) was subjected to SDS-PAGE and subsequently transferred to a PVDF membrane. Subsequently, the samples were incubated in TBST containing 5% skim milk for two hours. The primary antibody was then applied overnight at 4 °C, followed by a one-hour incubation at room temperature with the secondary antibody. The samples were then exposed to an ECL substrate, imaged, and documented.

### Real-Time quantitative Polymerase Chain Reaction (RT-qPCR)

Trizol reagent is employed for the extraction of total RNA from cells, with subsequent assessment of its purity and concentration conducted through the utilization of a micronucleic acid assay. The cDNA was synthesized in accordance with the instructions provided in the reverse transcription kit. The cDNA was then employed as a template to construct the reaction system for amplification, following the instructions set out in the fluorescence quantification kit. The expression was evaluated using the 2^−ΔΔCt^ method with U6 as the internal reference standard.

### Flow cytometry to detect apoptosis

The cells should be collected, subjected to centrifugation and then resuspended in PBS. Subsequently, the cells were stained using Annexin V-FITC and PI in accordance with the instructions provided. The extent of apoptosis was evaluated through flow cytometry following incubation.

Gating strategy: 1) Debris exclusion: Cells were gated on FSC-A vs. SSC-A to exclude debris (Gate P1). 2) Single-cell selection: FSC-H vs. FSC-A was used to eliminate doublets (Gate P2). 3) Viable cell gate: PI-negative cells were selected from Gate P2 to exclude dead cells (Gate P3). 4) Apoptosis analysis: Annexin V-FITC vs. PI fluorescence was analyzed on Gate P3. Quadrant gates were defined using unstained and single-stained controls: Annexin V-/PI-: Viable cells; Annexin V+/PI-: Early apoptotic cells; Annexin V+/PI+: Late apoptotic/necrotic cells; Annexin V-/PI+: Necrotic cells (if present).

Fluorescence compensation was performed with single-stained controls. Data analysis used FlowJo v10.8.

### In vivo tumor models

Nude mice, 5-weeks-old, were subcutaneously implanted in the right flank with 5 × 10^6^ stably transfected cells. Tumor growth was monitored weekly and harvested 5-weeks after implantation, weighed, and used for H&E staining and immunohistochemical staining.

### Dual luciferase reporter (DLR) assay

Luciferase reporter plasmids were transfected into appropriate cells, after which a dual-luciferase reporter assay kit (Beyotime, RG027) was utilized for determination of luciferase activity.

### Online database analysis

With “Naspharyngeal Carcinoma” as the keyword filtering in the GEO database (https://www.ncbi.nlm.nih.gov/geo/) to the relevant important data set of micrornas GSE32960 and GSE36682 data sets. The gene expression profiles of the above datasets were obtained using the “GEOquery” package and the differential expression maps were drawn using the “ggplot2” package. Wilcoxon test was used for statistical analysis. The rest of the database is used with ENCORI (https://rnasysu.com/encori/index.php), MiRWalk (http://mirwalk.umm.uni-heidelberg.de/) and miRmap (https://mirmap.ezlab.org/).

### Statistical methods

Results are reported as means ± SE and were compared in SPSS 26.0 (IBM, USA) using t-tests and ANOVAs; *p* < 0.05 was significant.

## Results

### miR-199a-5p is differentially expressed in NPC and regulates cisplatin resistance

**Fig. 1:**
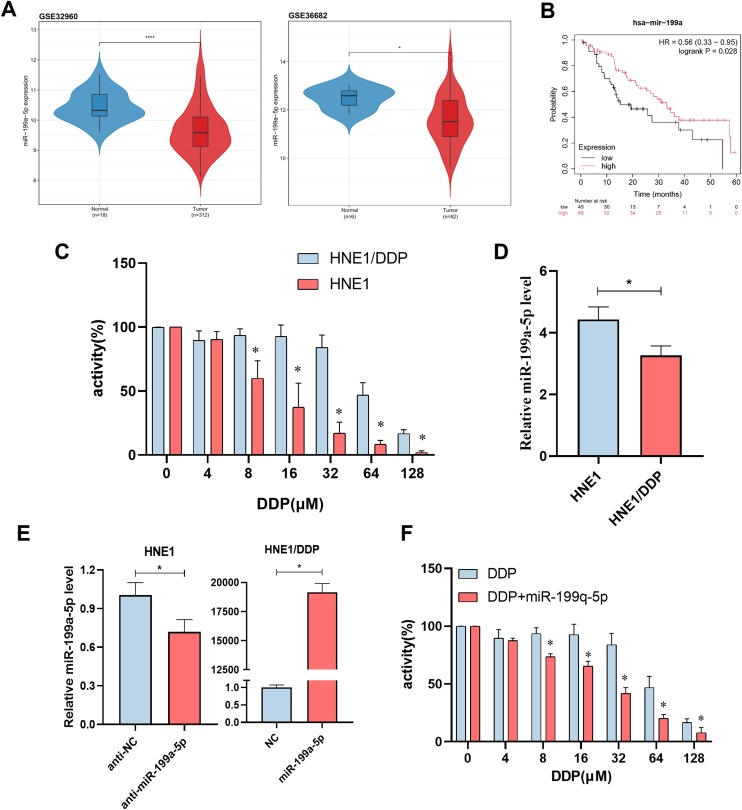
miR-199a-5p is differentially expressed in NPC and regulates cisplatin resistance. (A) The GSE32960 and GSE36682 datasets showed the expression of miR-199a-5p in NPC and normal tissues. (B) Overall survival of patients with different levels of miR-199a-5p was studied by Kaplan-Meier curve. (C) Study on the activity of two kinds of nasopharyngeal carcinoma cells under different DDP concentrations. (D) Expression of miR-199a-5p in two NPC cells. (E) The miR-199a-5p levels in the transfected cells were detected by RT-qPCR. (F) Regulation of HNE1/DDP cisplatin sensitivity by miR-199a-5p. Data are means ± SD, * *p* < 0.05.

In GSE32960 and GSE36682 databases, miR-199a-5p was identified to be differentially expressed in NPC and downregulated in tumor tissues (Fig. 1A). Subsequently, we found that there was a certain correlation between it and prognosis, and patients with high expression had a significantly better prognosis (Fig. 1B). Therefore, we further speculated whether miR-199a-5p expression would be down-regulated in cisplatin resistant patients with poor prognosis. Based on this, we compared the non-resistant cell HNE1 with the cisplatin-resistant cell HNE1/DDP, and the drug resistance of HNE1/DDP cells has been verified. The IC50 values of drug-resistant strain (HNE1/DDP) and non-drug-resistant strain (HNE1) were 61.63 μM and 11.53 μM, respectively, and the drug resistance index was 5.35(61.63 μM/11.53 μM), as shown in Fig. 1 C. It was verified that the expression of miR-199a-5p in HNE1/DDP cells was significantly lower than that in HNE1 cells (Fig. 1D).

After transfection (Fig. 1E), CCK-8 assay was used to investigate the effect of miR-199a-5p on cisplatin resistance (Fig. 1F). The results showed that the anti-tumor effect of DDP was more obvious in HNE1/DDP cells up-regulated by miR-199a-5p.

### miR-199a-5p inhibits the proliferative activity of NPC

**Fig. 2:**
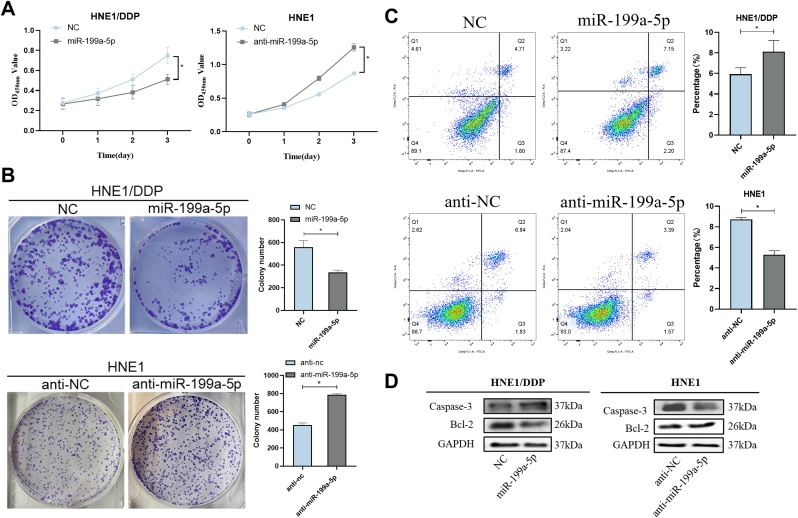
Effect of miR-199a-5p on the proliferation of NPC cells. (A) CCK8, (B) Cell cloning was used to detect the effects of different miR-199a-5p levels on proliferation of HNE1 and HNE1/DDP cells. (C) Effects of different levels of miR-199a-5p on apoptosis of HNE1 and HNE1/DDP cells. (D) Western immunoblotting was used to study the effect of miR-199a-5p on apoptotic proteins. Data are means ± SD, * *p* < 0.05.

To verify the regulatory role of miR-199a-5p in NPC, its inhibition of proliferation was tested. The results of CCK-8 assay and colony formation assay showed that miR-199a-5p could significantly inhibit the proliferation of NPC cells (Fig. 2 A and B). In the flow cytometry assay, we found that miR-199a-5p could significantly promote the apoptosis of NPC cells (Fig. 2C). In the subsequent detection of apoptosis-related proteins, our study showed that miR-199a-5p significantly increased the expression of Caspase-3 and decreased the expression of Bcl-2 (Fig. 2D). These results suggested that miR-199a-5p could inhibit the proliferation of NPC cells.

### miR-199a-5p inhibits the invasive and migratory activity of NPC

**Fig. 3:**
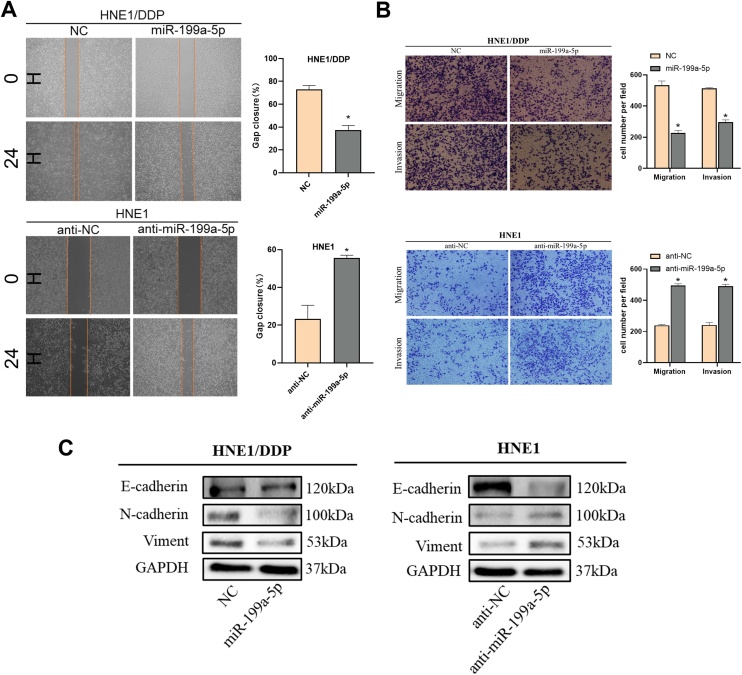
Effect of miR-199a-5p on migration and invasion. (A) Effects of different levels of miR-199a-5p on the migration of nasopharyngeal carcinoma cells. (B) Transwell assay was used to analyze the effect of miR-199a-5p on the migration and invasion of NPC cells. (C) Effect of miR-199a-5p on EMT-related proteins. Data are means ± SD, * *p* < 0.05.

In the wound-healing assay, our study showed that miR-199a-5p significantly inhibited the migration ability of NPC cells, while its knockdown increased the migration ability (Fig. 3A). Transwell assay also showed that miR-199a-5p significantly inhibited the migration of NPC cells. Invasion assay showed that miR-199a-5p could significantly inhibit the invasion ability of NPC (Fig. 3B). When we examined the expression of EMT-related proteins, the results were equally surprising. The expression of interstitial markers N-cadherin and Viment could be inhibited by miR-199a-5p and the expression of epithelial marker E-cadherin was promoted (Fig. 3C). These results suggested that miR-199a-5p enhanced migration and invasion by promoting EMT.

### miR-199a-5p can inhibit the growth of subcutaneous transplanted tumor in nude mice

**Fig. 4:**
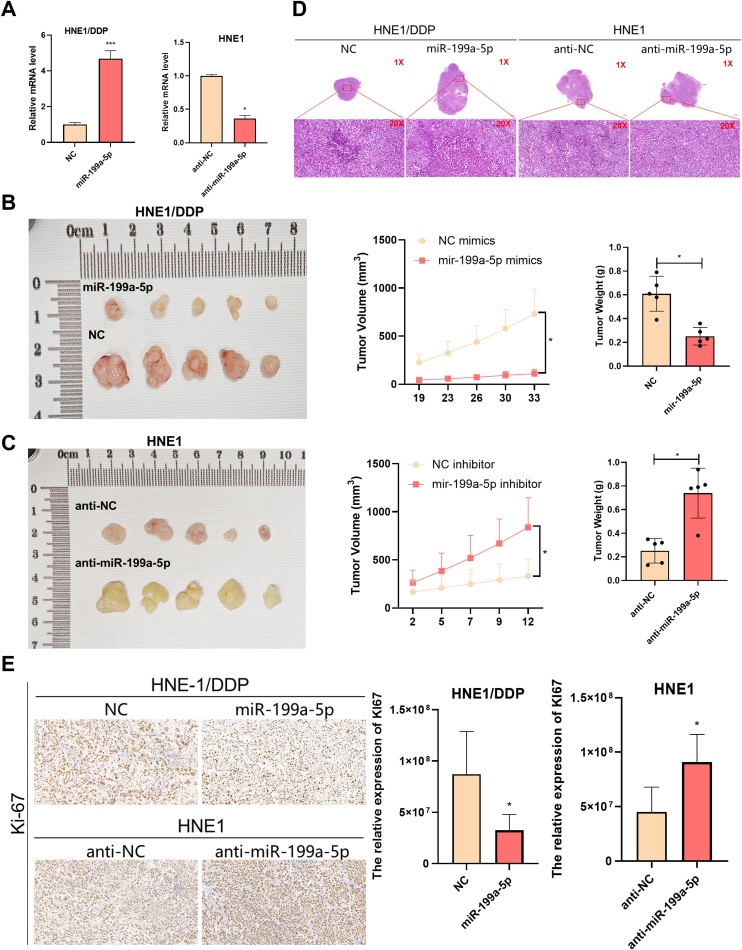
In vivo experiments confirmed the effect of miR-199a-5p on NPC. (A) Detection of lentivirus transfection efficiency in NPC cells. (B and C) Effect of miR-199a-5p on subcutaneous xenograft tumor in nude mice. (D) The tumors in nude mice were stained with H&E staining. (E) The expression of Ki-67 was detected by IHC. Data are means ± SD, * *p* < 0.05.

To further investigate the effect of miR-199a-5p on the malignant phenotype of NPC, animal experiments were performed. After lentivirus infection (Fig. 4A), the results of subcutaneous xenograft tumor experiments showed that miR-199a-5p could significantly inhibit tumor growth, on the contrary, knockdown of miR-199a-5p could promote tumor growth, and the tumor volume and weight were shown in Fig. 4 B and C. In the HE is staining of the tumors, we found that miR-199a-5p could significantly reduce the number of tumor cells, the cytoplasm and nucleus were lightly stained, and some of them showed karyolysis and cell disintegration (Fig. 4D). The expression of Ki-67 measured by IHC showed that miR-199a-5p reduced the expression of Ki-67, indicating that high miR-199a-5p expression is generally associated with lower malignancy (Fig. 4E).

### miR-199a-5p interacts with SLC1A5

**Fig. 5:**
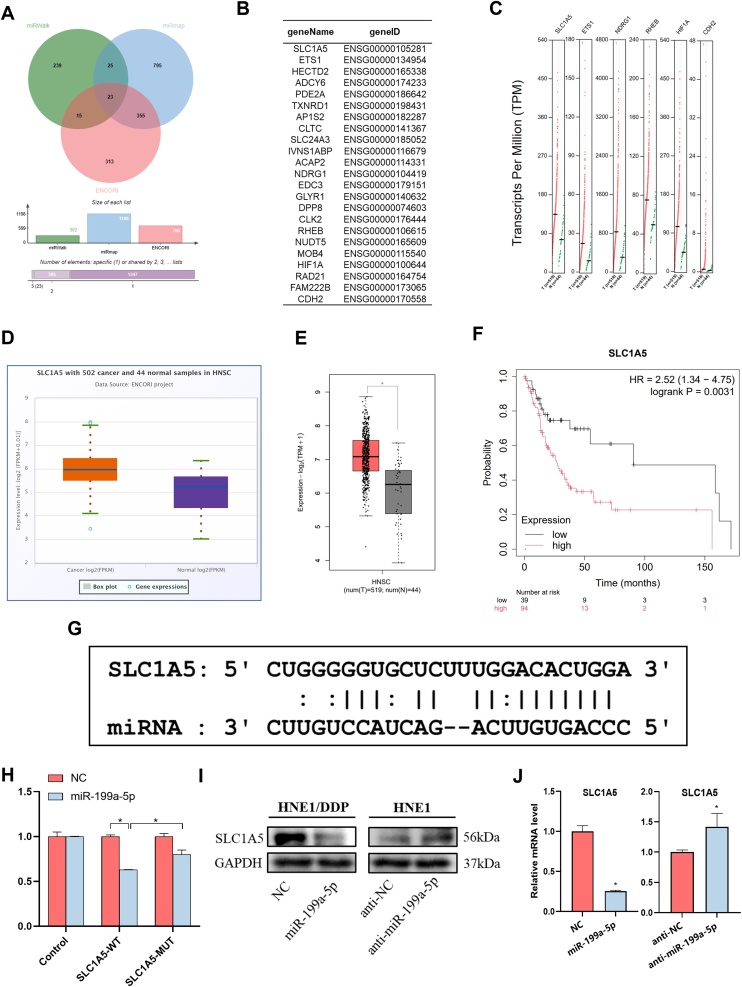
Targeting of SLC1A5 by miR-199a-5p. (A) Target analysis of miRWalk, miRmap, and ENCORI online databases. (B) Potential target genes were obtained by online database analysis. (C) Differentially expressed and up-regulated genes among the potential target genes. (D and E) Differential expression analysis of SLC1A5 in ENCORI and GEPIA2 online databases. (F) The effect of SLC1A5 expression level on the prognosis of patients. (G) Potential binding sites of miR-199a-5p and SLC1A5. (H) DLR analysis was used to verify the target between SLC1A5 and miR-199a-5p. (I‒J) Effect of miR-199a-5p on SLC1A5 levels by (I) Western immunoblotting and (J) RT-qPCR. Data are means ± SD, * *p* < 0.05.

In order to study the underlying mechanism of miR-199a-5p, the target of Mir-199a-5p was further explored. A total of 23 potential target genes were identified by searching in miRWalk, miRmap and ENCORI databases (Fig. 5 A and B). Among them, 6 genes were highly expressed in head and neck cancers, namely SLC1A5, ETS1, NDRG1, RHEB, HIF1A and CDH2 (Fig. 5C). Further analysis revealed that SLC1A5 has been shown to be a target gene of miR-199a-5p in thyroid cancer.^10^ Subsequently, using ENCORI and GEPIA2 online databases, we found that SLC1A5 showed significantly higher expression in HNSC (Fig. 5 D and E). Interestingly, higher SLC1A5 expression was associated with worse prognosis in female patients but not in male patients (Fig. 5F).

**Fig. 6:**
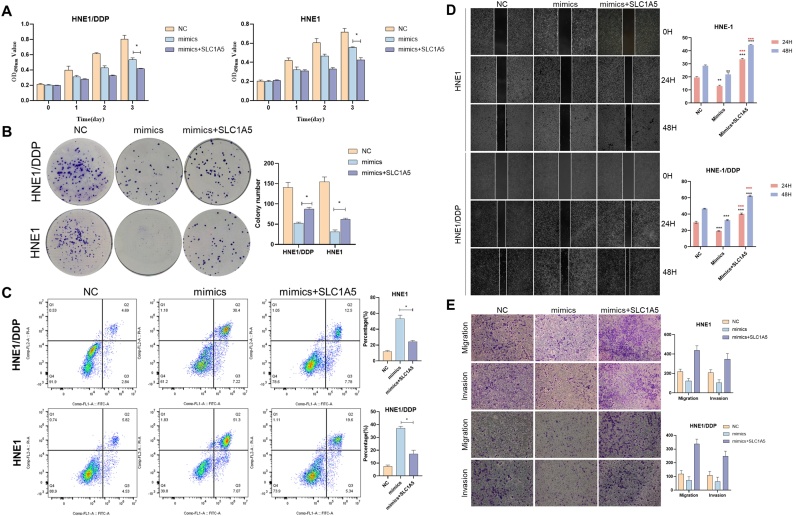
miR-199a-5p regulates the migration and invasion of nasopharyngeal carcinoma by inhibiting SLC1A5. CCK-8 assay (A) Clone formation assay (B) To assess the proliferative capacity of nasopharyngeal carcinoma cells; (C) Flow cytometry to detect the apoptotic ability of nasopharyngeal carcinoma cells; (D) Scratch test assesses the migration ability of nasopharyngeal carcinoma cells; (E) Transwell assay evaluates the migration and invasion capabilities of nasopharyngeal carcinoma cells, * *p* < 0.05.

The DLR assay was employed to substantiate the binding interaction between miR-199a-5p and SLC1A. The results demonstrated that the overexpression of miR-199a-5p markedly diminished the luciferase activity of SLC1A5-WT cells by 37% in comparison to the control cells. Following the SLC1A5 mutation, the luciferase activity in the SLC1A5-mut group exhibited a notable increase of 30.1% in comparison to the previous measurement (Fig. 5 G and H). The results of WB and RT-qPCR experiments demonstrated that the expression level of miR-199a-5p in NPC cells exerted a significant impact on the expression of SLC1A5 (Fig. 5 I and J). These findings suggest that miR-199a-5p directly targets SLC1A5 and regulates its expression (Fig. 6).

## Discussion

As previously mentioned, miRs play a crucial role in various physiological processes and are closely linked to a wide range of diseases. Numerous studies have shown their ability to regulate the translation of downstream target genes, thereby controlling organismal functions. Dysregulation of miR expression is strongly implicated in the development and progression of diverse diseases. For example, liver fibrosis can be influenced by serum exosome-derived miR-193a-5p and miR-381-3p through modulation of the AMPK/TGF-β/Smad2/3 signaling pathway,^11^ while lung cancer patients with significantly reduced levels of miRNA-508-3p tend to have poorer survival rates.^12^ In our investigation, we observed significant downregulation of miR-199a-5p in cisplatin-resistant nasopharyngeal carcinoma cells (HNE1/DDP). Through a series of phenotypic experiments, we successfully demonstrated that miR-199a-5p inhibits proliferation, invasion, and migration, and promotes apoptosis in nasopharyngeal carcinoma cells.

Numerous studies have provided compelling evidence supporting the significant role of EMT in tumor metastasis, inflammation, and various fibrotic diseases. Within the context of cancer, EMT is considered a crucial biological process that facilitates invasion, metastasis, and drug resistance.^13^^,^^14^ The primary function of E-cadherin lies in regulating intercellular adhesion and preserving the structural integrity and functionality of epithelial tissue. Notably, its expression undergoes a substantial reduction during EMT occurrence. Conversely, N-cadherin expression mediates the transition from epithelial to mesenchymal tissues in tumors, thereby enhancing tumor invasion.^15^ Vimentin primarily maintains cell shape as well as cytoplasmic and cytoskeletal integrity; its increased expression also characterizes EMT.^16^ Our study demonstrated that inhibition of miR-199a-5p resulted in the upregulation of N-cadherin and Vimentin while downregulating E-Cadherin levels in HNE1 cells. This suggests that anti-miR-199a-5p could potentially promote the development of EMT. Subsequent investigations revealed that miR-199a-5p can reverse the process of EMT in HNE1/DDP cells. Therefore, we can confidently affirm that miR-199a-5p exerts regulatory effects on EMT while inhibiting extracellular matrix degradation to reduce tumor migration and invasion rates.

Given the rapid proliferation of tumours, there is a necessity for them to take up a substantial quantity of Glutamine (Gln),^17^, ^18^, ^19^ and extracellular Gln can only enter the cell through a series of amino acid transporters on the cell membrane. It can be inferred that tumor tissues may facilitate Gln transport through the high expression of amino acid transporters. Solute Carrier Family 1 Member 5 (SLC1A5) is a significant member of the amino acid transporter family, and a substantial body of evidence indicates that SLC1A5 exhibits markedly elevated expression in a range of cancers.^20^, ^21^, ^22^, ^23^, ^24^ Some studies have shown that glutamine metabolism has an impact on tumor proliferation, invasion, and stimulation of tumor neovascularization.^25^^,^^26^ SLC1A5 be a downstream target of miR-145-5p to influence the progression of non-small cell lung cancer.^27^ Meanwhile, high SLC1A5 expression abolished the inhibitory effect of miR-125a-5p in renal cell carcinoma.^28^ Our study revealed that SLC1A5 is a downstream target of miR-199a-5p, and its high expression was found to be significantly associated with the development of NPC cells. Additionally, a correlation was observed between SLC1A5 expression and NPC drug resistance.

In our investigation, we observed a significant increase in the expression of the SLC1A5 gene in HNE1 cells when anti-miR-199a-5p was introduced. Conversely, there was a notable decrease in SLC1A5 expression levels in HNE1/DDP cells upon transfection with miR-199a-5p. These findings suggest that modulation of SLC1A5 expression by miR-199a-5p may potentially impede the proliferation, invasion, and metastasis of nasopharyngeal carcinoma cells.

## Conclusion

The high expression of miR-199a-5p can inhibit the malignant progression of nasopharyngeal carcinoma and enhance the sensitivity of NPC to cisplatin resistance. However, these effects are directly exerted by targeting SLC1A5. This study provides a new target for the diagnosis and treatment of NPC, and also brings new hope for patients with cisplatin-resistant NPC.

## Funding

This work was supported by the Natural Science Research Project of Anhui Educational Committee (2023AH051976); Bengbu Medical College's innovative training Program for postgraduate students under Grant (Byycx23029); and Project of Anhui Provincial Key Laboratory of Digital Medicine and Smart Health under Grant (AHCM2023Z003).

## ORCID IDs

Anchi Sun: 0009-0007-9554-544X; Rongrong Lv: 0009-0008-5053-6299; Zhiwei Xing: 0009-0007-4876-8528; Yifan He: 0009-0000-6203-0107; Xiaomin Wang: 0009-0005-1508-6534.

## Declaration of competing interest

The authors declare no conflicts of interest.
